# Psychometric Characteristics of the Italian Version of the Revised Sociosexual Orientation Inventory

**DOI:** 10.1007/s10508-024-02882-w

**Published:** 2024-06-12

**Authors:** Giacomo Ciocca, Roberto Giorgini, Laura Petrocchi, Giulia Origlia, Giuseppe Occhiuto, Antonio Aversa, Marco Tullio Liuzza

**Affiliations:** 1 Section of Sexual Psychopathology, Department of Dynamic and Clinical Psychology, and Health Studies, Sapienza University of Rome, Rome, Italy; 2 Department of Medical and Surgical Sciences, Magna Graecia University of Catanzaro, 88100 Catanzaro, Italy; 3 Department of Health Sciences, Magna Graecia University of Catanzaro, Catanzaro, Italy; 4 Department of Experimental and Clinical Medicine, Magna Graecia University of Catanzaro, Catanzaro, Italy

**Keywords:** Sociosexuality, Sociosexual Orientation Inventory, Evolutionary psychology, Psychometrics

## Abstract

Sociosexuality refers to the tendency to engage in uncommitted sexual behavior and has been dissected into three domains: sociosexual behavior, attitudes, and desire (Penke & Asendorpf, 2008), which led to the revised Sociosexual Orientation Inventory (SOI-R), which was validated on a German sample. The current research aimed at translating and validating an Italian version (I-SOI-R), administered to three distinct Italian participant groups. In the first sample (*N* = 710, females = 521, age = 18–59 years), we found evidence for a bifactor model, articulated in a general sociosexuality factor and three specific factors (behavior, attitudes, desire). High internal consistency was established for total and subscale scores, alongside favorable test–retest reliability. A connection was found between relationship status and sociosexual desire, though not gender dependent. We found evidence for test–retest reliability in a second sample (*N* = 55, females = 37, age 20–58 years). In a third study (*N* = 305, females = 147, age = 19–60 years), the earlier findings were replicated, further confirming the I-SOI-R's construct, criterion, and nomological validity on an online sample. Combining data from the three studies revealed full configural, metric, and scalar invariance regarding gender. This allowed us to meaningfully compare the observed scores of women and men and replicated the finding that men display higher levels of unrestricted sociosexuality. In conclusion, the I-SOI-R may serve as a valuable tool to assess and enhance sexual health, albeit warranting future research on construct and criterion validity.

**Supplementary Information:**

The online version contains supplementary material available at 10.1007/s10508-024-02882-w.

## Introduction

Sociosexuality is the tendency to engage in sexual behavior, generally but not necessarily, without a committed relationship or an affective bond. In other words, sociosexuality could be simply described as sex without love. From an evolutionary perspective, sociosexuality may increase the reproductive rate in men (Hallam et al., 2018) because, in the male population, fitness is mainly related to the quantity and number of partners, although this orientation is also present in women. In this regard, there exist asymmetries in parental investment, which lead to higher potential benefits and lower costs of unrestricted (or short-term mating) sociosexuality for men versus women (Buss & Schmitt, 2019; Gangestad & Simpson, 2000; Trivers, 1972). Considering the central role of sociosexual orientation for sexual, reproductive, and social health, the study of sociosexuality is aimed at the investigation and the prevention of sexually risky behaviors, above all, in an era where a large part of social relationships begins on the social network or the dating application (Ciocca, et al., 2020). For these reasons, the assessment of sociosexuality assumes a clinical and social meaning in the contemporary era.

The Sociosexual Orientation Inventory (SOI) was first developed by Simpson and Gangestad (1991) as a self-report tool to evaluate the sociosexual attitude and the tendency to more or less unrestricted sexual relationships. This psychometric tool comprised five items and was widely used in many research articles on this field (Simpson & Gangestad, 1991). However, the original version of SOI, with a sole global score, had some important weaknesses, such as the variable internal consistency of the SOI across samples, the low psychometric quality of the SOI, because of the open response formats of the first three items, the different methods of scoring of the SOI, and the sole inclusion of romantic couples in the original study by the SOI authors (Penke & Asendorpf, 2008).

Subsequently, Penke and Asendorpf (2008) developed a revised version of this tool, with an addition of further five items to improve the psychometric characteristics. The validity of the revised version of the SOI (SOI-R) was supported by (1) its factorial structure and (2) its criterion validity in terms of correlations with other expected sociosexual outcomes; prediction of one's own objectively coded sexual behavior toward an opposite-sex stranger; and changes in the romantic relationship during the year following the administration of the questionnaire (Penke & Asendorpf, 2008). Currently, the SOI-R remains the most widely used in psychological research.

The SOI-R is a self-report questionnaire that includes nine items and investigates three main dimensions of sociosexuality. The sociosexual behavior subscale (*α* = 0.85) examines the number of sexual partners with whom the subject has had emotionally disengaged relationships (e.g., “With how many different partners have you had sexual intercourse on one and only one occasion?”). The Sociosexual Attitude subscale (*α* = 0.87) measures the personal evaluation of emotionally disengaged sexual relationships (e.g., “I do not want to have sex with a person until I am sure that we will have a long-term, serious relationship.”). Finally, the sociosexual desire subscale (*α* = 0.86) assesses the frequency with which the subject expresses the desire to have a sentimentally disengaged sexual relationship, referring to a period of about one month (e.g., “How often do you experience sexual arousal when you are in contact with someone you are not in a committed romantic relationship with?”). The responses are given on a Likert scale from one to nine, although the response labels ranged from “0” to “20 or more” in the sociosexual behavior subscale, from “strongly disagree” to “strongly agree” in the sociosexual Attitude subscale and from “never” to “at least once a day” in the sociosexual desire subscale (Penke & Asendorpf, 2008).

Sociosexuality, as measured by the SOI-R, appears to mediate levels of psychological well-being following a causal relationship in terms of self-esteem and relationship satisfaction, decreasing depressive and anxiety symptomatology (Vrangalova & Ong, 2014). Furthermore, high levels of satisfaction have been found in individuals with a sociosexual orientation engaged in consensually non-monogamous (CNM) relationships (Rodrigues et al., 2017). Greater sexual freedom would affect perceptions of infidelity and the likelihood of engaging in unfaithful behavior. It has been seen that higher sociosexuality, i.e., unrestricted sociosexuality, correlates positively with greater concern about the sexual infidelity of the partner rather than emotional infidelity. Similarly, high levels of sociosexuality would increase individuals' likelihood of being unfaithful toward their partners (Blomkvist et al., 2021; Mattingly et al., 2011; Rodrigues & Lopes, 2017; Rodrigues et al., 2017; Weiser et al., 2018).

Although it is not possible to define sociosexuality as the main cause of individual distress per se (Dubé et al., 2017), several studies highlight important relationships between sociosexuality and psychological distress. Indeed, unrestricted sociosexuality is positively related to depressive and anxiety symptoms, especially in women (Grello et al., 2003; Regenerus & Uecker, 2011), to Dark personality traits (Machiavellianism, narcissism, and psychopathy; Jonason & Buss, 2012; Jonason et al., 2009, 2012; Koladich & Atkinson, 2016; Stolarski et al., 2017), insecure attachment styles (Brennan & Shaver, 1995), and risky behaviors (Corbin et al., 2016; Furman & Collibee, 2014; Hall & Pichon, 2014; Testa & Hone, 2019; Townsend et al., 2020). Studies on attachment theory suggest that secure attachment styles are predictors of narrower sociosexuality (Szepsenwol et al., 2017). In contrast, avoidant and ambivalent attachment styles are positively associated with high unrestricted sociosexuality (Sprecher, 2013). Moreover, some sociodemographic aspects, such as religiosity and political orientation, were studied in several investigations about sociosexuality (Barrada et al., 2018; Claxton & van Dummen, 2013; Neto, 2016; Penke & Asendorpf, 2008).

In conclusion, the SOI-R in the study of the psychological variables involved in sociosexuality has proved to be very useful. In this regard, the instrument was validated in Hungarian (Meskó et al., 2014), Portuguese (Nascimento et al., 2018; Neto, 2016), and Spanish (Barrada et al., 2018; Romero et al., 2023).

Therefore, developing an Italian version of this instrument could implement the knowledge of how sociosexuality manifests itself in the population. However, although an Italian translation of the SOI-R is available online, an Italian version of the SOI-R has never been adequately validated. The present work aims to provide translation, administration, and validation of the Italian version of the Revised Sociosexual Orientation Inventory.

In a first study on a convenience sample of predominantly young female adults, we aimed at exploring the structural validity of the SOI-R, and to estimate its reliability in terms of internal consistency. On top of that, we sought to replicate Penke and Asendorpf's findings on the interaction between the SOI-R subscale, gender, and relationship status. We expected to find lower levels of sociosexual desire among people in a relationship (vs. singles), especially among men (vs. women). In a second study on a smaller sample, we tested the SOI-R reliability in terms of temporal stability.

In a third study on a more diverse and gender-balanced sample, we aimed to replicate the results from study one, and we tested measurement invariance conditional on gender, but also to establish criterion validity, construct validity, and nomological validity.

## Studies 1–2

### Method

#### Participants

The translated Italian version of the SOI-R (henceforth I-SOI-R) was administered to 713 participants (females = 524, males = 179, M age = 26.62 years ± 6.22 SD, min = 16, max = 59) from an online platform through snowball recruitment on the popular social media outlets (Facebook and Instagram). Another, independent, smaller sample (*N* = 55, females = 37, M age = 26.6 years ± 6.94 SD, min = 20, max = 58) completed the survey twice, at time zero and after 14 days, to compute the test–retest reliability of the Italian translation of the SOI-R. Data were collected from January to March 2021. Descriptive statistics of our sample are reported in Tables 1, 2. Data from three participants were excluded from the analysis because their reported age displayed age was lower than 18.

**Table 1:** Descriptive statistics of the demographic variables from Study 1

		Age			Education		Relationship		Sexual orientation	
*N*		M	SD	Max Min	M	SD	Single	In a relationship	Exclusively heterosexual	Other
Combined	710	26.32	6.20	59 18	3.16	0.97	306 (43.09%)	404 (56.90%)	575 (80.98%)	135 (19.01%)
Females	521	26.55	6.18	58 18	3.15	0.91	204 (39.15%)	317 (60.84%)	431 (82.72%)	90 (17.27%)
Males	179	26.87	5.90	54 18	3.19	1.13	96 (53.63%)	83 (46.37%)	144 (80.44%)	35 (19.35%)

Education’s range: 1–6

**Table 2:** Descriptive statistics of the demographic variables of the dataset from Study 2

		Age			Education		Relationship (*t*0)		Relationship (*t*1)	
N		M	SD	Max Min	M	SD	Single	In a relationship	Single	In a relationship
Combined	55	26.6	6.93	58 20	2.05	0.70	21 (38.18%)	34 (61.81%)	19 (34.54%)	36 (65.45%)
Females	37	26.55	6.18	54 22	1.91	0.43	13 (35.13%)	24 (64.86%)	12 (32.43%)	25 (67.56%)
Males	18	26.87	5.90	58 20	2.33	1.02	8 (44.44%)	10 (55.55%)	7 (38.88%)	11 (61.11%)

Education’s range: 1–4

### Measures and Procedure

#### Sociosexual Orientation Inventory Translation

After getting permission from the authors who developed the scale, we translated the original version of the SOI through a forward and backward procedure. The translation and adaptation from English to Italian were conducted by two expert bilingual translators and a team of psychometricians, clinical psychologists, and sexologists, who evaluated each item's translation, taking comprehensibility into account.

#### Demographic Variables

We collected the following sociodemographic variables: age (in years), gender (0 = “Female,” 1 = “Male”), education (1 = “Primary school,” 2 = “Secondary school,” 3 = “Bachelor degree,” 4 = “Master degree,” 5 = “Ph.D./Medical specialization”), relational status (0 = “single,” 1 = “in a relationship”), and sexual orientation (which was dichotomized as follows: 0 = “exclusively heterosexual, 1 = “non exclusively heterosexual”). Table 3 shows the zero-order correlations among the variables measured in Study 1.

**Table 3:** Zero-order correlations

Variable	M	SD	1	2	3	4	5	6	7	8
1. Age	26.64	6.11								
2. Education	3.16	0.97	.28**							
3. Gender	0.26	0.44								
4. Sexual Orientation	0.18	0.38								
5. Relationship	0.57	0.50								
6. Behavior	2.47	1.45	.03	.01	.16**	.17**	−.15**			
7. Attitude	5.69	2.14	.01	.02	.16**	.11**	−.06	.47**		
8. Desire	3.62	1.91	−.01	−.06	.34**	.12**	−.38**	.40**	.39**	
9. SOI TOT	3.93	1.44	.01	−.01	.28**	.16**	−.25**	.75**	.82**	.77**

Gender, *M* = 1, *F* = 0; Sexual orientation, 0 = Exclusively heterosexual, 1 = Other; Relationship single = 0, In a relationship = 1; *SOI TOT* = Sum score/*n* item of the three SOI subscales

### Data Analysis

We realized that we mistakenly used a 10-point format in the first three items, separating the response “20” from “or more.” Therefore, we collapsed the responses to points 9 and 10 for these first three items. After recording reverse keyed items (item 6), we submitted our data to the following statistical analyses.

#### Confirmatory Factor Analysis

We tested the structural validity of the I-SOI-R through confirmatory factor analysis (CFA). For this purpose, we built three models and compared the fit indices of each model to identify the best-fitting one.

The Mardia test for multivariate normality showed that our data were not normally distributed both in terms of skewness (1961.23*, p* < 0.001) and kurtosis (16.35, *p* < 0.001). Therefore, we used a maximum likelihood robust (MLR) estimator.

The three models included the original unidimensional structure of SOI (Simpson & Gangestad, 1991) and the three-factor structure proposed by Penke and Asendorpf (2008). Finally, a bifactor model modified the three-factor model by adding a general factor reflected by all the items. We estimated different fit indices for all models, which included: chi-squared (χ^2^) Comparative fit index (CFI), Tucker Lewis index (TLI), Root Mean Squared Error of Approximation (RMSEA), and Standardized Root Mean Squared Residual (SRMR). Cutoff values for fit were considered adequate if CFI and TLI were > 0.90, RMSEA < 0.06, and SRMR < 0.08 (Hu & Bentler, 1999). Since we used an MLR estimator, we reported the robust versions of CFI, TLI, and RMSEA. Furthermore, as recommended by recent literature (Bonifay et al., 2015; Rodriguez et al., 2016), we tested whether our bifactor model was essentially unidimensional. To this purpose, we computed: (1) the percentage of uncontaminated correlations (PUCs)—the percentage of correlations that reflect the variance of the general factor (Bonifay et al., 2015; Reise et al., 2013)—and (2) the explained common variance (ECV)—the percent of common variance that is attributed to the general factor in the bifactor model (Reise et al., 2010), which is a measure of the degree of essential unidimensionality (Rodriguez et al., 2016; Sijtsma, 2009). Following the recommendations from Reise et al. (2013), values of *ω*h > 0.70, ECV > 0.60, and PUCs > 0.80 indicate a strong general factor, suggesting that a unidimensional model specification should not lead to biased estimates.

#### Reliability

Reliability was tested regarding temporal stability (test–retest reliability) and internal consistency. Reliability of temporal stability was computed on a sample of 55 participants (18 M, M age = 26.6 years ± 6.94 SD) who completed the survey twice, thus allowing for testing test–retest reliability, although we selected and tested at the first administration 100 subjects. Considering the asymmetric distribution of the SOI-R scores, we used Spearman’s rho (*ρ*) rank correlation coefficients to estimate the association between scores at T0 and T1.

Reliability in terms of internal consistency was assessed by computing the omega total (⍵_t)_ and the hierarchical omega (⍵_h_) and was computed based on the best CFA model. We chose omega (McDonald, 1999) because it has less restrictive assumptions than the more popular Cronbach’s alpha (Sijtsma, 2009; Triano-Hermosilla & Alvarado, 2016). Omega only assumes a congeneric measurement model, namely a model where factor loadings are free to differ. Another advantage of the omega coefficient is that it is well suited for addressing reliability in bifactor models, allowing the partition of the total reliability (omega total) into reliability in measuring the general factor (hierarchical omega) and reliability of the specific (group) factors (omega group).

#### Gender Differences

Nomological validity was tested by comparing mean differences between males and females, as previous findings and theoretical considerations predict higher levels of restricted sociosexuality in women compared to men.

#### Interaction Between Relationship Status and Gender

Although we did not collect information on the duration of the romantic relationships, we collected recorded participants’ relationship status. Therefore, we sought to replicate Penke and Asendorpf's (2008) findings on the interaction between the SOI-R subscale, gender, and relationship status. We expected to find lower levels of sociosexual desire among people in a relationship (vs*.* singles), especially among men (vs. women). This hypothesis was tested through an analysis of covariance (ANCOVA) controlling for age, which is a variable that has previously been found to be related to sociosexuality, even though with mixed results (e.g., Meskó et al., 2014; Neto, 2016; Penke & Asendorpf, 2008). For this purpose, we fitted a multilevel linear model with participants and items as random intercepts. The ANCOVA was tested through a type III Wald–χ^2^ test to compare deviance across different models.

All the analyses were conducted performed in the statistical programming environment R (R Core Team, 2021), using the following packages: *lavaan* (Rosseel, 2012), *semTools* (Jorgensen et al., 2021), *semplot* (Epskamp, 2019), *psych* (Revelle, 2021), *effects* (Fox, 2003), *sjplot* (Lüdecke, 2021), *admisc* (Dusa, 2022), *compareGroups* (Subirana, Sanz & Vila, 2014), *ggplot2* (Wickham, 2016), *rstatix* (Kassambara, 2021), *emmeans* (Lenth, 2022), *tidyr* (Wickham & Maximilian, 2022), *lme4* (Bates et al., 2015), *lmerTest* (Kuznetsova et al., 2017), and *buildmer* (Voeten, 2022).

## Results

### Confirmatory Factor Analysis Results

The one-dimensional model in which all items loaded onto a single factor was not supported by data (*χ*^2^ = 951.81, df = 27,* p* < 0.001, CFI = 0.65 TLI = 0.53, RMSEA = 0.22, SRMR = 0.12).

The three-factor model, in which Items 1–3 loading on “sociosexual behavior,” Items 4–6 on “sociosexual attitude,” and Items 7–9 on “sociosexual desire,” fit the data well (*χ*^2^ = 66.31, df = 24, *p* < 0.001, CFI = 0.98, TLI = 0.98, RMSEA = 0.050, SRMR = 0.04).

**Fig. 1:**
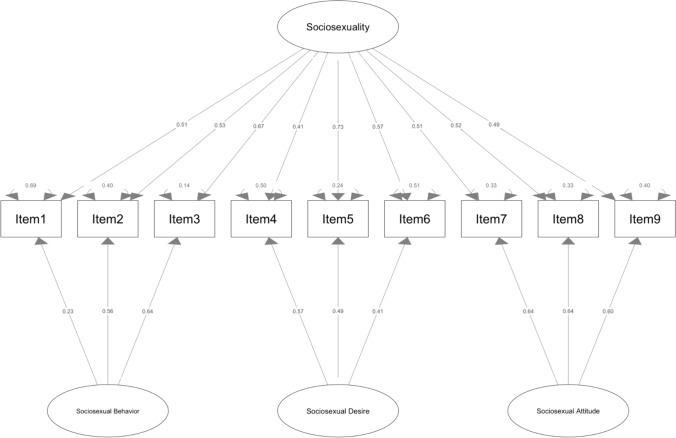
Path diagram of the bifactor model. Path coefficients are standardized

The last model, the bifactor (see Fig. 1), where the structure is the same of three-factor except that all items load also on a general “Sociosexuality” factor, was the best one (*χ*^2^ = 40.26, df = 18, *p* = 0.002, CFI = 0.99, TLI = 0.98, RMSEA = 0.041, SRMR = 0.025).

### Internal Consistency and Essential Unidimensionality

We found excellent reliability of the I-SOI-R total score (*⍵*_*t*_ = 0.91, *⍵*_*h*_ = 0.68), and good reliability for the subscales (0.85 ≥ *⍵*_*t*_*s* ≥ 0.80) in terms of internal consistency. Values of PUCs and ECV for the bifactor model were, respectively, 0.75 and 0.51 and did not reach the suggested cutoffs (PUCs > 0.80 and ECV > 0.60) to consider the SOI as essentially unidimensional, considering that *⍵*_*h*_ < 0.70. We found that the I-SOI-R had excellent test–retest reliability as a total score and as single subscales (*ρs* ≥ 0.72, *ps* < 0.001).

### Gender Differences

**Fig. 2:**
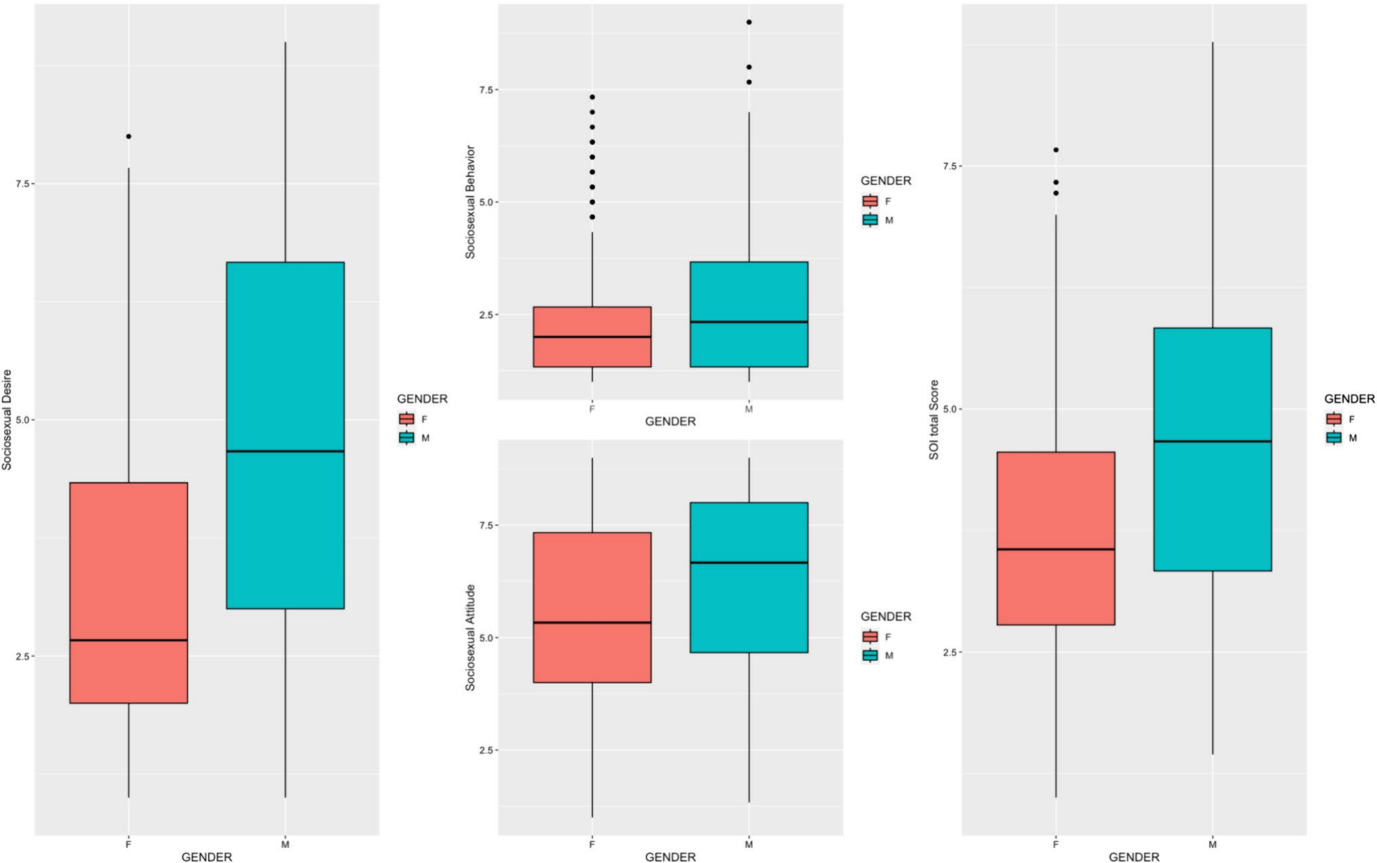
SOI scores distribution across genders. The y-axis represents the adjusted means of each group controlling for age

No significant differences were observed in age and education between males and females (Age, *t* = − 0.61, *df* = 698, *p* = 0.54; Education, *t* = − 0.59, df = 698, *p* = 0.60). We tested whether the relationship status impacted the I-SOI-R and whether this effect interacted with the subscale of the I-SOI-R and gender. We found an interaction between the subscales and relationship (*χ*^2^ $=$ 99.16, df = 2, *p* < 0.001). Indeed, singles displayed greater levels of sociosexual desire (*t* = 10.65, df = 590.44, *p* < 0.001, Cohen’s *d* = 0.83) and sociosexual behavior (*t* = 3.8, df = 545.66, *p* < 0.001, Cohen’s *d* = 0.30) but this effect of relationship status was not found for sociosexual attitude (*t* = 1.69, df = 644.4, *p* = 0.09, Cohen’s *d* = 0.13). The interaction between subscale, gender, and relationship status was not significant (*χ*^2^ $=$ 4.93, df = 2, *p* = 0.08). Importantly, we also found an interaction between gender and the I-SOI-R subscale (χ^2^ $=$ 14.93, df = 2, *p* < 0.001), which is explained by the fact that although men displayed significantly higher levels of sociosexuality across all the subscales (ts ≥ 4.18, df = 698, ps < 0.001), the effect was more marked in the desire subscale (Cohen’s *d* = 0.83) as compared to other subscales (Cohen’s *d*s $\le$ 0.38) (Fig. 2).

## Discussion

This first study provided compelling evidence in favor of the validity and reliability of the Italian version of the SOI-R. We confirmed the hypothesized factorial structure of the SOI-R on an Italian sample, namely the items reflected the three dimensions of behavior, attitudes, and desire.

However, the good support for a bifactor structure allowed us also to partition the reliability in the reliability in measuring the general factor, showing that attitudes seem to be the most related to the general factor, whereas behavior appears to be the most specific domain. Overall, the reliability of the I-SOI-R total scores and its subscales in terms of internal consistency was good to excellent.

Furthermore, we also found good support for the reliability of the I-SOI-R total scores and its subscales in terms of temporal stability on a subsample. Indeed, the data collected in Studies 1–2 came from a convenience sample that consisted predominantly of young female students. On top of that, the results at hand do not provide sufficient evidence for the construct validity of the scale.

For these reasons, we decided to conduct a third study on a more diverse sample using the platform Prolific. In this survey, we included measures that assessed mainly overlapping constructs (sexual disgust and sexual desire) and related but distinct constructs (hypersexuality) to assess convergent and discriminant validity, respectively. Furthermore, we added measures of self-reported sexual behavior (e.g., frequency of masturbation) aimed at establishing criterion validity. On top of that, other measures such as religiosity (Barrada et al., 2018) and personality measures (Nascimiento et al., 2018) were included because they are theoretically related to sociosexuality and thus serve the purpose of establishing nomological validity for our instrument. A more diverse sample also allowed us to test whether the I-SOI-R works in the same way among females and males (measurement invariance conditional on gender).

## Study 3

### Method

#### Participants

For this study, we created an online questionnaire on Google Forms, which was then administered through the Prolific online platform to recruit 400 Italian participants. Sixteen participants could not terminate the survey because they failed the attention check. From the remaining 305 responses, we excluded a further 10 participants, as they reported different nationalities than Italian. Each participant also received a fee of approximately 1.74 euros. The final sample was therefore composed of 295 (females = 141, M age = 29.71 years ± 8.54 SD, min, min age = 20, max age = 60, see Table 4).

**Table 4:** Descriptive statistics of the demographic variables of Study 3

	*N*	Age				Education		Relationship			
		Mean	SD	Max	Min	Mean	SD	Single	R1P	RMP	OR
Combined	295	29.71	8.54	60	20	14.99	2.35	135	148	3	9
								(46%)	(50%)	(1%)	(3%)
Females	141	29.87	9.29	60	20	15.13	2.27	58	76	3	4
								(41%)	(54%)	(2%)	(3%)
Males	154	29.56	7.81	58	20	14.87	2.42	77	72	0	5
								(50%)	(47%)	(0%)	(3%)

Religiosity		Living area		Pol. orient.		Masturb.		Npartners		NSOTC	
Mean	SD	Urban	Rural	Mean	SD	Mean	SD	Mean	SD	Mean	SD
2.56	2.13	225	70	3.74	1.85	5.73	2.14	3.79	2.31	1.66	1.66
		(76%)	(24%)								
2.53	2	109	32	3.53	1.82	4.64	2.14	3.89	2.33	1.69	1.77
		(77%)	(23%)								
2.58	2.26	116	38	3.92	1.87	6.73	1.59	3.69	2.30	1.63	1.56
		(75%)	(25%)								

Education’s range: 8–21; Religiosity’s range: 1–10; Pol. Orient.: 1–10; Masturbation’s rage: 1–9; N partners’ range: 1–9; and NSOTC’s range: 1–9

After providing informed consent, participants were allowed to quit the study at any point. We used a questionnaire to collect some sociodemographic data, including age, gender, education level, religion, relationship status, and residence.

Two items were used regarding gender: the first for the sex observed at birth (male, female, intersex), and the second for the gender they identified with (male, female, genderqueer, intersex, other). Regarding education level, the six options available ranged from elementary school to doctorate. Relationship status was investigated using four possible answers: single, stable relationship with a partner, stable relationship with multiple partners, and casual relationships. Autoeroticism was also investigated through the frequency of masturbation, with nine possible answers: from “never” to “at least once a day.” Regarding political orientation, we used two items: in the first asking to place oneself on a 10-point scale going from “left” to “right” and in the second from “conservative” to “progressive.” Finally, excluding the region of residence, we focused on the area of residence by dividing it into “urban” and “rural.”

We also included an attention check item that consisted of a multiple-choice question. The text of the question was as follows: “Most modern theories of decision-making take into account the fact that decisions are not made in a vacuum. Factors such as individual preferences and knowledge, along with situational variables, can have a strong impact on the decision-making process. To facilitate our research on decision-making, we are therefore interested in obtaining more information about some factors that concern it. In particular, we are interested in knowing if you take the time to read the survey instructions. Otherwise, some of our manipulations may be ineffective, as they are based on changes to the instructions. So, to demonstrate that you have read the instructions, please select “Other.” Thank you very much.” The subject would then have to select the answer “Other” (and write something about it, according to the Google Forms settings) among eight other answers such as: “reading,” “watching a film,” “religious activities,” “card games or a play,” “cooking,” “gardening,” and “traveling.” Participants who would have answered differently than “Other” would have been excluded. Another exclusion criterion was the non-Italian nationality of the participants. Unfortunately, we had to discard the information relating to the “region of residence” due to an error in the drafting of the questionnaire.

#### Measures and Procedure

The study was conducted online through Google Forms. All participants completed the SOI-R, along with other measures. To assess construct validity, we used three additional measures: the Italian version of the Hypersexual Behavioural Inventory (HBI, Ciocca et al., 2020); the “sexual” dimension of the Three Domains of Disgust Scale (TDDS, Tybur et al., 2009); and the Italian version of Sexual Desire Inventory-2 (SDI-2, Callea & Rossi, 2021).

The HBI consists of 19 items rated on a 5-point Likert scale with responses ranging from 1 (*never*) to 5 (*very often*), with higher scores indicating a major deficit in controlling impulses. Some examples of items are: “I use sex to forget the worries of everyday life,” or “My thoughts and my sexual fantasies distract me from performing important tasks.” The scale assesses the tendency toward hypersexual behavior through three subscales—one for each domain of hypersexuality—coping, control, and consequences.

The TDDS consists of 21 items rated on a 7-point Likert-type response format ranging from 0 (*not disgusting at all*) to 6 (*extremely disgusting*). This scale assesses disgust across three main domains: pathogen, sexual, and moral disgust. The sexual subscale was used for our study (e.g., “Engaging in anal sex with a person of the opposite sex,” “Perform oral sex”).

The SDI-2 is a 13-item (e.g., “When you spend time with an attractive person, how strong is your sexual desire?”, “How strong is your desire to engage in sexual behavior by yourself?”) that assesses dyadic and solitary sexual desires.

Finally, participants also completed a short personality questionnaire, the Italian version of Ten Item Personality Measure (TIPI, Jonason et al., 2011; validated in Italian by Chiorri et al., 2015) that is a “short” measure of the Big Five personality dimensions: openness (e.g., “open to new experiences, with many interests”), conscientiousness (e.g., “dependable, self-disciplined”), extraversion (e.g., “outgoing, exuberant”), agreeableness (e.g., “understanding, loving”), neuroticism (e.g., “argumentative, quarrelsome”). The test consists of 10 items rated on a 7-point Likert-type response format ranging from 1 (*strongly disagree*) to 7 (*strongly agree*).

Furthermore, religiosity was investigated through a single item (e.g., “How religious do you consider yourself?”) in a 10-point response format ranging from 1 (*not at all*) to 10 (*extremely*).

### Data Analysis

#### Confirmatory Factor Analysis (CFA)

After recording reverse keyed items (Item 6), we submitted our data to the following statistical analyses. We tested the structural validity of the I-SOI-R through CFA. For this purpose, we built three models and compared the fit indices of each model to identify the best one.

The Mardia test for multivariate normality showed that our data were not normally distributed both in terms of skewness (1512.11, *p* < 0.001) and kurtosis (17.61, *p* < 0.001). Therefore, as in Study 1, we used an MLR estimator. Since we used an MLR estimator, we reported the robust versions of CFI, TLI, and RMSEA and pursued the same analytical approach as described in Study 1. We also evaluated essential unidimensionality as described in Study 1. The scale's reliability based on the selected model was also evaluated through McDonald’s Omega based on the CFA model. See Study 1 for more details.

#### Construct Validity

Following the recommendation from Rönkkö and Cho (2022), we assessed discriminant validity with HBI by assessing the correlation between the latent variable of the general factor SOI and the latent factor of the HBI, and then testing whether the upper limit of the 95% confidence intervals (95% CI) was greater than 0.8. The measurement model for the HBI was modeled assuming that the measure is unidimensional.

#### Criterion Validity

For establishing criterion validity, we regressed the self-reported frequency of masturbation, number of partners (controlling for age), and sex outside of the couple (SOTC, controlling for age) on the SOI general factor. Since SOTC is a binary outcome, we modeled it as a categorical variable using the WLSMV estimator in *lavaan*. In case we found a positive relationship between the general SOI factor and SOTC, we followed this up by assessing whether there was also a linear relationship between the number of SOTC partners and the general factor of SOI, controlling for age. All the other variables were z-transformed to better interpret the strength of their relationship.

#### Criterion Validity

We investigated whether the general SOI factor correlated with the following measures: religiosity and political orientation (left–right and progressive–conservative).

Since the self-reported political ideology in terms of left–right was highly correlated with the self-reported ideology in terms of progressive–conservative (*r* = 0.69), we averaged the two items to have a unique indicator of self-reported political ideology.

We tested whether which of the personality traits computed from the TIPI predicted the general SOI factor total score and the scores from the subscales correlated with the personality domains measured by the TIPI.

Finally, we tested whether there was an interaction between gender and relationship controlling for age on the Desire subscale to see if we could replicate our results from Study 1. To test this hypothesis, we conducted an ANCOVA on the sum score of the Desire subscale with gender (males vs. females) and relationship (single vs. in a relationship) as factors, and age as a covariate. Even though people could also report being in a polyamorous relationship, or being involved in multiple occasional relationships, the frequency of the responses to these latter categories was very low (2, and 9, respectively). Therefore, we collapsed these two categories with “in a relationship” and “single,” respectively.

#### Gender Differences and Measurement Invariance (ME/I)

Nomological validity was tested by comparing mean differences between males and females, as previous findings and theoretical considerations predict higher levels of restricted sociosexuality in women compared to men. However, to compare mean differences between males and females in I-SOI-R scores, it is first necessary to ascertain that the I-SOI-R measures sociosexuality in the same way across genders, namely showing ME/I. When comparing groups, scholars should first ascertain whether the measurement has the same factor solution between groups (configural invariance), the same loading across groups (metric invariance), and the same intercepts across groups (scalar invariance). To compare the means of the I-SOI-R, scalar invariance should be met otherwise a mean difference in the means could not reflect real differences in the latent variable (LV) and, vice versa, a lack of difference in the observed scores could reflect a real difference in the LV). Therefore, we tested the invariance of measurement across the studied groups. If the *χ*^2^ difference between models was not significant, the more restrictive assumption did not lead to a significant decrease in fit. However, since this method is prone to reject MI even in the presence of trivial non-invariance (Hays et al., 2005), in case of significance we rejected MI when Delta CFI <−0.010, and Delta RMSEA > 0.015 or Delta SRMR > 0.030, when assessing metric invariance, and Delta CFI <−0.010, and Delta RMSEA > 0.015 or Delta SRMR > 0.010 when assessing scalar invariance (Chen, 2007).

Given the paucity of the sample, we tested ME/I on a bigger sample where we collapsed data from studies 1 and 3 (*N* = 1015, females = 657, M age = 27.55 years ∓ 7.12, min age = 18, max age = 60).

## Results

Table 4 shows the descriptive statistics for the demographic variables, and Tables 5, 6 show the descriptive statistics for the variables used in Study 3.

**Table 5:** Descriptive statistics of the Sociosexual Orientation Inventory (SOI) in Study 3

	M	SD	Skewness	Kurtosis
SOI 1	1.93	1.08	2.61	9.83
SOI 2	1.81	1.43	2.65	7.30
SOI 3	2.13	1.90	1.84	2.43
SOI 4	6.47	2.40	-0.85	-0.32
SOI 5	4.38	2.72	0.25	-1.28
SOI 6	4.35	2.62	0.30	-1.18
SOI 7	4.09	2.23	0.35	-1.09
SOI 8	3.01	1.97	0.95	-0.17
SOI 9	2.95	2.00	0.92	-0.38

SOI’s range: 1–9

**Table 6:** Descriptive statistics of the items of the Hypersexual Behaviour Inventory (HBI), Three Domains of Disgust Scale (TDDS), and the Sexual Desire Inventory (SDI), Study 3

	M	SD	Skewness	Kurtosis
HBI 1 HBI2	2.18 2.03	1.20 1.22	0.73 0.90	−0.53 −0.42
HBI 3 HBI4	2.11 1.53	1.20 0.97	0.80 2.01	−0.46 3.36
HBI 5 HBI 6	1.44 2.00	0.79 1.16	1.76 0.90	2.21 −0.24
HBI7 HBI8	1.85 2.04	1.07 1.19	1.16 0.82	0.55 −0.50
HBI 9 HBI10	1.63 1.34	0.93 0.76	1.44 2.47	1.38 5.96
HBI 11 HBI 12	1.47 1.44	0.94 0.87	2.13 2.17	3.85 4.23
HBI 13 HBI 14	2.79 1.32	1.24 0.66	0.01 2.47	−1.08 7.07
HBI 15 HBI 16	1.72 1.83	1.04 1.11	1.45 1.23	1.27 0.51
HBI 17 HBI 18	1.38 1.54	0.79 0.91	2.37 1.72	5.66 2.12
HBI 19 TDDS 1	1.38 2.34	0.71 1.67	2.29 0.39	6.11 −0.59
TDDS 2 TDDS 3	1.07 0.94	1.57 1.46	1.64 1.77	1.97 2.54
TDDS 4 TDDS 5	2.29 1.80	1.90 1.88	0.36 0.87	−1.03 −0.39
TDDS 6 TDDS 7	3.90 1.97	2.29 2.07	−0.60 0.63	−1.19 −1.00
SDI 1 SDI 2	3.82 3.44	2.01 2.23	−0.23 −0.11	−0.79 −1.10
SDI 3 SDI 4	5.18 3.33	2.12 2.21	−0.68 0.21	−0.23 -0.99
SDI 5 SDI 6	3.92 5.50	2.27 1.97	−0.04 −0.87	−1.03 0.29
SDI 7 SDI 8	5.44 5.43	1.93 2.15	−0.80 −0.89	0.17 0.09
SDI 9 SDI 10	3.74 3.63	2.08 2.04	0.04 −0.32	−0.58 −0.94
SDI 11 SDI 12 SDI 13	4.38 4.74 4.02	2.17 2.36 2.22	−0.41 −0.60 −0.17	−0.65 −0.66 −0.66

HBI’s range: 1–5; TDDS’s range: 0–6; SDI’s range: 0–7

### Confirmatory Factor Analysis

The bifactor model was confirmed to be the best-fitting mode, as compared to the tridimensional model (*Δχ*^2^ = 22.62, df = 6, *p* < 0.001) which fitted better than the unidimensional model (*Δχ*^2^ = 231.65, df = 3, *p* < 0.001). Furthermore, the bifactor model is confirmed to have excellent fit indices (CFI = 0.99, TLI = 0.99, RMSEA = 0.034, SRMR = 0.027).

### Internal Consistency

We found that the reliability analysis demonstrated excellent reliability of the I-SOI-R total score of the I-SOI-R *(⍵*_*t*_ = 0.93, *⍵*_*h*_ = 0.71), and good reliability for the subscales (0.86 ≥ *⍵ts* ≥ 0.80). Values of PUCs and ECV for the bifactor model were, respectively, 0.75 and 0.50 and did not reach the suggested cutoffs (PUCs > 0.80 and ECV > 0.60) to consider the SOI as essentially unidimensional, considering that *⍵*_*h*_ < 0.70.

### Construct, Criterion, and Nomological Validity

#### Construct Validity

We found that the relationship between the general factor of the SOI and the factor of the HBI significantly correlated (*β* = 0.37, *SE* = 0.10, *z* = 3.6, *p* < 0.001), but the upper limit of the 95% CI (0.57) fell well below the cutoff considered as problematic (0.8) by Rönkkö and Cho (2022). Therefore, even though the measurement of hypersexual behavior is related to SOI, they tap into separate constructs.

When assessing the correlation between the general factor of the SOI and the factor of the TDDS-sex, we found a high negative correlation (*β* = − 0.65, SE = 0.58, *z* = − 11.26, *p* < 0.001). The lower limit of the 95% CI (− 0.75) fell well below the cutoff considered problematic |0.8|.

We found that the relationship between the general factor of the SOI and the factor of the SDI significantly correlated (*β* = 0.61, SE = 0.06, *z* = 10.15, *p* < 0.001), but the upper limit of the 95% CI (0.72) fell well below the cutoff considered as problematic (0.8). However, since SDI should specifically tap into the desire aspect of the SOI, we repeated the same analysis, but by modeling only two unidimensional measurement models (one for the SOI desire subscale, and one for the SDI), and looked at the correlation between the two factors. We found that the relationship between the factor of the SOI-Desire and the factor of the SDI significantly correlated (*β* = 0.48 SE = 0.07, *z* = 7.42, *p* < 0.001), but the upper limit of the 95% CI (0.61) fell below the |0.8| cutoff.

#### Criterion Validity

The SOI general factor predicted the number of lifetime partners (*β* = 0.76, SE = 0.05, z = 16.87, *p* < 0.001), the frequency of masturbation (*β* = 0.39, SE = 0.08, *z* = 4.85, *p* < 0.001), and sex outside of the couple (*β* = 0.62, SE = 0.07, *z* = 8.53, *p* < 0.001).

To quantify the effect size of the last effect, the difference in the total observed SOI score between people who reported to have experienced SOTC versus people who did not is a Cohen’s *d* = 0.92, which is deemed as a large one. Furthermore, we found that the SOI general factor predicted the number of SOTC partners among people who reported to have had SOTC (*β* = 0.78, SE = 0.2, *z* = 3.80, *p* < 0.001).

#### Other Relevant Predictions

The general factor SOI was negatively (*β* =−0.15, SE = 0.07, *z* = − 2.08, *p* = 0.04) related to religiosity. A negative significant relationship was found also with political ideology *β* = − 0.16, SE = 0.08, *z* = − 2.00, *p* = 0.04).

#### Relationship with Personality

When regressing the SOI general factor onto the five personality dimensions measured by the TIPI, we found that the SOI general factor was significantly and positively predicted by openness (*β* = 0.28, *SE* = 0.08, *z* = 3.64, *p* < 0.001) and, negatively, by agreeableness (*β* = − 0.19, SE = 0.08, *z* = − 2.50, *p* = 0.01) and by neuroticism (*β* = − 0.18, *SE* = 0.08, *z* = − 2.26, *p* = 0.02). However, we did not find an independent significant effect for either extraversion (*β* = 0.09, SE = 0.1, *z* = 0.94, *p* = 0.35) or conscientiousness (*β* = − 0.09, SE = 0.09, *z* = − 1.07, *p* = 0.29).

#### Interaction Between Relationship Status and Gender

We replicated the results of Study 1, as we only found a main effect of gender (*F*(1, 279) = 29.05, *p* < 0.001)—with male participants displaying greater levels of sexual desire than women—a main effect of being in a relationship (*F*(1, 279) = 13.09, *p* < 0.001)—with singles displaying greater levels of sexual desire than people in a relationship. However, we did not find any significant interaction (*F*(1, 279) = 1.01 *p* = 0.31).

#### Overall Confirmatory Factor Analysis

The three subscales displayed medium-to-large correlation coefficients (0.38 $\le$ Pearson’ *r*s $\le$ 0.45). The Mardia test for multivariate normality showed that our data were not normally distributed both in terms of skewness (3091.40, *p* < 0.001) and kurtosis (23.16, *p* < 0.001). Therefore, as in Study 1, we used an MLR estimator. Since we used an MLR estimator, we reported the robust versions of CFI, TLI, and RMSEA and pursued the same analytical approach as described in Study 1. We also evaluated essential unidimensionality as described in Study 1.

The bifactor model was confirmed to be the best-fitting model, as compared to the tridimensional model (*Δχ*^*2*^ = 35.52, df = 6, *p* < 0.001), which fitted better than the unidimensional model (*Δχ*^*2*^ = 1215.90, df = 3, *p* < 0.001). Furthermore, the bifactor model is confirmed to have excellent fit indices (CFI = 0.99, TLI = 0.99, RMSEA = 0.037, SRMR = 0.021).

#### Overall Internal Consistency

We found that the reliability analysis demonstrated excellent reliability of the I-SOI-R total score of the I-SOI-R (*ω*_*t*_ = 0.91, *ωh* = 0.69), and good reliability for the subscales (0.85 ≥ *ωts* ≥ 0.81). Values of PUCs and ECV for the bifactor model were, respectively, 0.75 and 0.50 and did not reach the suggested cutoffs (PUCs > 0.80 and ECV > 0.60) to consider the SOI as essentially unidimensional, considering that ⍵h > 0.71.

#### Measurement Invariance Conditional on Gender

After excluding 16 participants who did not identify either as female or male, we conducted our multi-group measurement invariance analysis on the remaining 994 participants (657 females). We found that configural (CFI = 0.99, TLI = 0.99, RMSEA = 0.03, SRMR = 0.02), full metric invariance (Δ*χ*^*2*^ = 38.12 df = 14, *p* < 0.001, but ΔCFI >—0.010), and scalar invariance held (*Δχ*^*2*^ = 46.052, df = 5, *p* < 0.001, ΔCFI = − 0.010, ΔRMSEA < 0.15 and ΔSRMR = 0.10). Therefore, we could meaningfully compare the observed scores across genders to make inferences about gender differences in the latent variables.

#### Gender Differences

We found that men exhibited significantly higher levels of unrestricted sociosexuality than women in the total I-SOIR-R observed score (*t* = 7.11, df = 992, *p* < 0.001, Cohen’s *d* = 0.48), in the social attitudes (*t* = 4.33, df = 992, *p* < 0.001, Cohen’s *d* = 0.29), and in the desire subscale (*t* = 10.2, df = 992, *p* < 0.001, Cohen’s *d* = 0.68). However, men and women did not differ significantly in the behavior subscale (*t* = 1.51, df = 992, *p* = 0.13, Cohen’s *d* = 0.1). These results show that the effect sizes vary substantially, from the negligible and non-statistically significant difference in sociosexual behavior to the medium-to-large effect in sociosexual desire. However, on top of comparing men and women through the univariate effect size, it is also important to understand how they vary globally (Del Giudice, 2009, 2022; Eagly & Revelle, 2022). Therefore, to assess global differences between women and men, we also used their differences in the three subscales to compute the Mahalanobis distance *D*, a multivariate generalization of Cohen’s *d* that can be used as a standardized effect size for multivariate differences between groups. We found that a Mahalanobis distance *D* = 0.78 which confirms that the effect is large.

## Discussion

The results from Study 3 replicate and extend the results from Study 1, as the bifactor structure of the scale was confirmed, thus allowing for the use of the I-SOI-R as a higher-order construct, articulated in different manifestations (behavior, attitudes, desire). The overall CFA and measurement invariance conducted on the whole sample further corroborated these findings and showed that scalar invariance for gender groups was found. This implies that research on gender differences in the SOI could meaningfully compare observed scores. In our study, we found medium-to-large gender differences, as men showed higher levels of unrestricted sociosexuality as compared to women, a result that is consistent with the established literature (Schmitt, 2005), although the overall effect size found in our study (Cohen’s *d* = 0.48) is smaller than the one found by Schmitt (2005, Cohen’s *d* = 1.16), which was based on the old unidimensional SOI measure (Simpson & Gangestad, 1991). Also, a multivariate effect size (Mahalanobis’ *D* = 0.78) provides evidence for a much larger effect size.

Results from Study 3 provided compelling evidence for its construct validity in the Italian population. We found that the I-SOI-R, although correlated with hypersexuality, sexual desire, and sexual disgust, is theoretically and empirically discernable from these constructs. The finding about hypersexuality was expected, since sociosexuality is a conscious variant of sexual behavior, whereas sociosexuality is a disorder related to the psychopathology of behavioral addictions. The finding about sexual disgust seems to be partially in contrast with what was reported in previous studies where sexual disgust and SOI-R were treated as manifestations of the same latent variable (Izzo et al., 2022). However, the study from Izzo and colleagues was conducted on a much smaller sample and did not have the power to conduct latent variable models aimed at addressing this research question with sufficient precision.

Study 3 also provided criterion validity by showing that the general factor underlying the I-SOI-R was related to relevant behavior such as the number of lifetime partners, frequency of masturbation, and sex with extra-dyadic partners. Unsurprisingly, the general factor underlying the I-SOI-R was also negatively related to religiosity and political ideology, a finding that is consistent with the previous literature (Barrada et al., 2018; Claxton and van Dummen, 2013; Neto, 2016; Penke & Asendorpf, 2008).

Finally, our results on the relationship between the general factor underlying the I-SOI-R and the big five personality traits as measured by the TIPI showed that, when partialling out the variance shared by the five dimensions, the best positive predictor of unrestricted sociosexuality was openness to experience. This result, although theoretically expected, has not been consistently found in previous cross-cultural research (Schmitt & Shackelford, 2008). We also found that people who are lower in neuroticism and agreeableness were more likely to have higher levels of unrestricted sociosexuality. While the finding on agreeableness has been found, although not consistently, in previous cross-cultural studies (Schmitt & Shackelford, 2008), the finding on neuroticism is somewhat more surprising considering the extant literature. Even more surprising, in our study, was that extraversion did not independently predict levels of unrestricted sociosexuality, a result that seems to be well-established in the literature (Nascimiento et al., 2018; Schmitt & Shackelford, 2008). However, it must be noted that there are some notable differences between this study and the previous literature on personality and sociosexuality. For starters, previous studies mostly used the first version of the SOI (Simpson & Gangestad, 1991), rather than the one revised by Penke and Asendorpf (2008). Secondly, previous studies typically looked at zero-order correlations, whereas we looked at the incremental predictivity of each personality trait when controlling for the variance shared with other traits. Finally, we assessed this relationship by looking directly at the general factor estimated within a bifactor model that partials out the variance specifically accounted for by the specificity of the subscale (behavior, attitudes, desire). Also, our analytical approach removes the measurement error shared by the items.

## General Discussion

Although sociosexuality had been traditionally defined and measured as a global tendency to uncommitted sex, Penke and Asendorpf (2008) showed that three distinct components characterize this construct: sociosexual behavior, sociosexual desire, and sociosexual attitude. They devised a revised version of the former SOI-R based on this observation.

Hence, the Italian version of SOI-R (I-SOI-R) represents a fundamental and pivotal tool in researching sexual behavior for several reasons concerning clinical and prevention issues. Sexual health is strongly related to sociosexual behavior, and some investigations have found a relationship between sexual risk behavior and sociosexuality (Sevi et al., 2018; van Dijk et al., 2021).

In the present study, we translated and validated an Italian version of the SOI-R (I-SOI-R) and administered it to two different samples to cross-validate our results from the first sample, which was made mostly of college students, to a more diverse sample recruited online. Our CFA analysis of the whole dataset showed that the best-fitting model was a bifactor model with sociosexuality as a general factor and behavior, desire, and attitude as specific factors. This result slightly differs from Penke and Asendorpf (2008) because even though it acknowledges the specificity of the three components of the SOI and cannot be considered as an essentially unidimensional measurement, it also identifies that the items also reflect a general factor. This finding supports the notion that sociosexuality can still be conceived as a general attitude, although with specific factors, a finding that lies in between the first conceptualizations of the SOI, and the revised scale proposed by Penke and Asendorpf. Also, the components seem to differ a bit in their specificity: Sociosexual desire seems to have the greatest specificity among the specific factors, as it displays the greater omega group (0.50), followed by sociosexual behavior (0.44), and sociosexual attitude (0.32).

We tested reliability both in terms of internal consistency and in terms of temporal stability. We found compelling evidence that the total score of this instrument has excellent reliability, and its subscales also display good internal consistency. We also found a good level of test–retest reliability for the I-SOI-R total scores and its subscales. To our knowledge, this is the first validation study that also assesses test–retest reliability, although on a limited subsample (see the limitations discussed below). We emphasize that internal consistency was assessed through the *omega* coefficients computed directly from the bifactor model. This approach has two strengths: (1) It does not assume a tau-equivalent measurement model, which is a mostly non-tenable assumption, and (2) it allows us also to evaluate the reliability of the general factor and the specific group reliability for the specific factors (Flora, 2020), thus providing a more nuanced picture of the specific contribution of each specific component, as described above.

We showed that the I-SOI-R is a measurement that is scalar invariant to gender, which allows meaningful comparisons between observed means of male and female participants. This analysis extends the validity of the measurement compared to Penke and Asendorpf (2008), who did not assess such a crucial prerequisite for meaningful group comparisons. Barrada et al. (2018), pursued a similar approach during the validation of the SOI-R in Spanish, although they also tested for measurement invariance by age.

Similar to Penke and Asendorpf (2008), we found gender differences in the I-SOI-R. Men display higher levels of unrestricted sociosexuality, a result that is consistent with evolutionary accounts of gender differences in mating strategies (Al-Shawaf et al., 2018; Buss & Schmitt, 1993). The effect is particularly pronounced (medium-to-large) for the Sociosexual Desire component, coherently with the previous literature concerning this facet of sexual behavior (Barrada et al., 2018; Penke & Asendorpf, 2008). Our results on the sexual behavior component are also in line with previous studies, which typically found negligible differences in the sexual behavior component (Penke & Asendorpf, 2008; while Barrada et al., 2018 found the difference in the opposite direction, with women slightly more unrestricted than men in this component). In our study, we also followed recent recommendations on how to report gender differences (Del Giudice, 2022; Eagly & Revelle, 2022), and provided a multivariate effect size, which shows that taken together, gender differences in the I-SOI-R are large and consistent with previous meta-analytic accounts (Archer, 2019).

Importantly, as discussed before, in our third study on an online sample, we provided further evidence of criterion validity, construct validity, and nomological validity. Most of our results resonate with previous literature, although we found a somewhat peculiar pattern of results when it comes to personality, as we found openness, but not extraversion, to be the personality trait that mostly predicts unrestricted sociosexuality. We discussed how these results might be related to the different analytical strategies pursued in the present validation study.

Differently from Penke and Asendorpf (2008), we also failed to replicate the interaction between gender and relationship status in affecting sociosexual desire. However, an important difference is that we did not track whether the present relationship lasted for more than four years, which is the time after which typically sociosexual desire increases, despite the ongoing long-term commitment (Fisher, 1987). On top of that, it is hard to draw causal conclusions on this effect. On the one hand, it might be that sociosexual desire decreases after starting a relationship. On the other hand, it might well be that people with low levels of sociosexual desire are more likely to commit to a romantic relationship.

The abovementioned consideration highlights the first and most important limitation of this study: Being cross-sectional in its design, it prevents us from establishing temporal precedence of one trait (sociosexual desire) on one behavior (being in a relationship). Such a claim could be supported by future longitudinal studies.

The second significant limitation of the present study is that it is based on a convenience sample, which essentially differs from the general Italian population in some demographic characteristics even though study 3 partially addressed this issue by reaching a higher proportion of male participants and the non-student population.

A third limitation is that we estimated test–retest reliability on a relatively small sample (*N* = 55) and, therefore, should be taken cautiously, although the results are reassuring.

Another limitation is that this validation study did not test more extensively the criterion validity of the SOI-R both in terms of concurrent validity (e.g., correlation with other behaviors that stem from promiscuous sociosexuality) and in terms of predictive validity (e.g., predicting changes in the romantic relationship), as in Penke and Asendorpf (2008).

However, we also want to stress that this study has some important strengths. First, we used a bifactor model to validate the I-SOI-R. Such a strategy has many benefits, such as being able to partition the reliability between the general factor and the group factors. Having found good evidence in favor of this model also allowed us to rethink sociosexuality as a general factor, even though it is articulated in different manifestations (behavior, attitude, desire). Second, the aforementioned approach allowed us to better explore the other aspects of the I-SOI-R validity looking directly at the general factor underlying this instrument. Third, this study investigated measurement invariance conditional on gender and found that we can meaningfully compare women and men in the I-SOI-R total and subscale scores.

### Future Directions

Future studies should fill this gap and target more diverse samples or, possibly, a nationally representative sample, also by testing these other aspects of the validity in Italian samples to corroborate the notion that the I-SOI-R could be confidently used to measure sociosexuality and its components (desire, behavior, and attitudes) in the Italian population. Therefore, from a clinical perspective, I-SOI-R can represent a useful tool to evaluate and promote sexual health, especially because of the frequent associations found in the literature between high levels of sociosexuality and risky sexual behavior. In addition, this instrument could also be useful in combination with other tests investigating psychological and personality aspects, which in the literature are often found in subjects with high levels of sociosexuality (e.g., anxiety, depression, insecure attachment, Dark traits personality).

### Conclusions

The present study validated the Italian version of the SOI-R (I-SOI-R) in a large Italian sample. The results suggest that the I-SOI-R may be used as a reliable and valid measure of global sociosexuality in the Italian population to prevent eventual attitudes toward sexually risky behavior.

### Supplementary Information

Below is the link to the electronic supplementary material.

Supplementary file1 (DOCX 14 KB)

## Data Availability

Data to reproduce the analysis are available on the open science framework repository: https://osf.io/5jqme/.

## Code Availability

The R scripts to reproduce the analyses are available on the open science framework repository: https://osf.io/69evs/.

## Material Availability

The I-SOI-R is available on the open science framework repository: https://osf.io/965eu/.
