# Supplementary material for: Psychometric Characteristics of the Italian Version of the Revised Sociosexual Orientation Inventory
Source: Arch Sex Behav. 2024 Jun 12;53(8):3267–83. doi: 10.1007/s10508-024-02882-w (PMC11335888; doi:10.1007/s10508-024-02882-w)
Supplement: Supplementary file 1 — Supplementary file1 (DOCX 14 KB) [file 10508_2024_2882_MOESM1_ESM.docx]

**Supplementary material**

**The I-SOI-R**

Si prega di rispondere sinceramente alle seguenti domande:

1. Con quanti partner diversi hai avuto rapporti sessuali negli ultimi 12 mesi?

0; 1; 2; 3; 4; 5-6; 7-9; 10-19; 20 o più

2. Con quanti partner diversi hai avuto un rapporto sessuale in una sola e unica occasione?

0; 1; 2; 3; 4; 5-6; 7-9; 10-19; 20 o più

3. Con quanti partner diversi hai avuto un rapporto sessuale senza avere interesse in una relazione seria e duratura con la persona?

0; 1; 2; 3; 4; 5-6; 7-9; 10-19; 20 o più

4. Sesso senza amore va bene.

1; 2; 3; 4 ; 5; 6; 7; 8; 9

In forte disaccordo Assolutamente d’accordo

5. Riesco ad immaginarmi a mio agio nel fare sesso occasionale con partner diversi.

1; 2; 3; 4; 5; 6; 7; 8; 9

In forte disaccordo Assolutamente d’accordo

6. Non voglio fare sesso con una persona se non sono sicuro/a che avremo una relazione seria e a lunga durata.

1; 2; 3; 4; 5; 6; 7; 8; 9

In forte disaccordo Assolutamente d’accordo

7. Quante volte ti capita di avere delle fantasie sessuali su una persona con cui non hai una relazione sentimentale seria?

1. Mai

2. Molto raramente

3. Circa una volta ogni due o tre mesi

4. Circa una volta al mese

5. Circa una volta ogni due settimane

6. Circa una volta alla settimana

7. Diverse volte a settimana

8. Quasi ogni giorno

9. Almeno una volta al giorno

8. Quante volte provi eccitazione sessuale quando sei a contatto con qualcuno con cui non hai una relazione sentimentale seria?

1. Mai

2. Molto raramente

3. Circa una volta ogni due o tre mesi

4. Circa una volta al mese

5. Circa una volta ogni due settimane

6. Circa una volta alla settimana

7. Diverse volte a settimana

8. Quasi ogni giorno

9. Almeno una volta al giorno

9. Nella vita quotidiana, quanto spesso hai fantasie sessuali spontanee su una persona che hai appena incontrato?

1. Mai

2. Molto raramente

3. Circa una volta ogni due o tre mesi

4. Circa una volta al mese

5. Circa una volta ogni due settimane

6. Circa una volta alla settimana

7. Diverse volte a settimana

8. Quasi ogni giorno

9. Almeno una volta al giorno
